# Dermatological Do-It-Yourself

**Published:** 1980

**Authors:** J. L. Burton

**Affiliations:** Consultant Dermatologist, Bristol Royal Infirmary


### Dermatological do-it-yourself: How to nail your neighbour's wife to the bedroom floor

**J. L. Burton**
Consultant Dermatologist, Bristol Royal Infirmary

It was my wife's fault, as usual. She insisted that I nail down the squeaking floorboards in our large senescent 'semi' on the first Saturday afternoon after we moved in. Anyone who can cope with the average dermatological out-patient clinic is inclined to make light work of knocking in a couple of thousand nails however, and I felt more than usually self-satisfied as I completed my labours and slouched downstairs for a well-earned beer. My efforts at household maintenance are rarely concluded so successfully, and my growing self-esteem was only slightly clouded by the fact that I couldn't find the beer. This small warning cloud on the horizon of my happiness quickly manifested itself in the most astonishingly physical way as a distinct spot of rain on my bald pate.

This struck me as peculiar since I was on the upper hall-landing at the time (maybe she's hidden the beer in the wardrobe), but being intellectually gifted, I quickly concluded that the roof must leak, as it often does in these old houses. Unusual though, on such a sunny day.

We scientific types can usually think of a variety of explanations for natural phenomena, and I quickly figured that the rain must somehow have been stored in the roof during yesterday's downpour, and had just percolated through. Strange though, that our surveyor hadn't commented on this structural fault. Also, I was on the second floor of a four-storey house.

Feeling more than usually like the proverbial plodding policeman, I received inspiration as the second large drop of water exploded on the aforementioned pate. Nails, floor-boards, water-pipes, burst. Don't panic, do something, keep cool, ask wife, where's wife? Shopping who knows where, for who knows how long. Alright, don't panic, any doctor can cope with emergencies. Turn the water off. Simple.

By now the spots which had increased in both rate and volume, were making rather a damp patch on the carpet. A rapid tour of the house revealed a rich variety of taps under sinks, in cupboards and craftily hidden behind curtains, and all these I firmly turned off. I then calmly awaited the cessation of the steady trickle, but unfortunately it continued to gain strength and the awful realisation gradually dawned on me that I was marooned in a sinking house. I didn't panic of course. Years of medical training stood me in good stead as I watched hair-line cracks appear in the plaster, and it became apparent that it was only a matter of time before tons of water would cascade in on me in a shower of plaster and undeleted expletives.

The sagging sub-epidermal blister which was rapidly developing in our ceiling clearly needed aspirating, and urgently. Resourceful as ever, I required only two more lightning tours of the house befofe I found a suitable hypodermic implement, a long-handled broom, hidden away in what my wife later claimed was a broom cupboard. Taking careful aim, I ran lightly across the landing, sprang gracefully upward in a double axel, a la Robin Cousins, and in a breathtaking poem of coordination and manual dexterity, I firmly jabbed the broom-handle into the apex of the crepitant bulla. I regained terra firma (or at least soggy hall carpet) bathed in triumph and about 0.4 seconds later I was bathed in several gallons of ice-cold water and assorted chunks of plaster.

It occurred to me as I splodged morosely downstairs that I had better get help. The subsequent telephone conversations I had with various plumbers' wives, just at the most exciting stages of the televised England v. Wales Rugby International, have been mercifully obliterated by those protective psychic mechanisms which prevent insanity, but I do recall that the lady with the most polite and cooperative husband relayed the message that he would definitely be prepared to consider the matter again first thing on Monday morning if things had not improved. It was then that I had my brain-wave. Semi-detached houses are built as symmetrical pairs, and therefore wherever my neighbour turned off his water supply, there turned I. Brilliant. Neighbour's wife answers phone, charming girl, most sympathetic, husband would have been delighted to help but unfortunately he and both their children were in the throes of a touch of the influenzal moribundity, but never mind, she'd come round herself. Marvellous creature, beautiful and efficient, arrived within seconds and immediately found the spot where they turned their water off. Unfortunately our tap was missing as the two houses were not in fact quite symmetrical.

By now the miniature waterfalls cascading gently down the stairs were producing the gentle susurration which one commonly associates with mountain rills. I was about to sit down and cry, when Mary brightly announced that we must rip up the bedroom floor-boards, locate the burst pipe and plug the hole with something until such time as a plumber could be cajoled or threatened to attend.

Ne'er did neighbours of the opposite sex race for the bedroom with such breathless anticipation, or on such short acquaintance. Having arrived, it took me only a few minutes to go back downstairs again to look for a crowbar. Mary meanwhile had assumed what in happier circumstances I would have construed as a most provocative posture, with her back to the doorway, her ear to the floor and her callipygous charms most beautifully displayed. There ensued five minutes of the most strenuous physical exertion as I manfully wielded my crowbar, tore aside the carpets and we heaved up the floor-boards together in a desperate struggle to reach the fountain-head. Freud would have loved it.

At last our efforts were rewarded, and as I tore up a length of wet planking, the offending nail was wrenched from the pipe and a great jet of water shot across the room and deluged the new wall-paper. Mary showed great presence of mind and immediately placed her thumb firmly over the hole, just as I allowed the slippery wet plank to escape from my grasp. Like a striking snake, the liberated plank whipped viciously back into place, and of course Mary's thumb was directly in the track of the hole-bound nail. I apologised of course. She went slightly pale, and generously pointed out that the flow of water had virtually ceased now that she was firmly nailed in place. We had a brief discussion over the relative merits of releasing her and leaving her 'in situ' while I fetched a plumber, but in the end my natural gallantry prevailed and I advised her to bite her lip as I hauled up the plank again.

This time the jet of liquid which sprayed onto the wallpaper was thinner but redder, and I realised that Mary had courageously but most unwisely left her thumb pressed tightly on the hole and the water was going through the perforation. It would be pleasant to record that at this stage I took firm charge of the proceedings, and organised the speedy repair of both unwanted orifices, but the sad truth is that Mary, bleeding gently, went off to fetch help while I performed a passable imitation of the little Dutch Boy. What blandishments she offered him I never learnt, but within minutes a very capable builder arrived, smirking somewhat. As it happened he also failed to find the appropriate tap, but at least he had the nous to find the grate in the road which concealed the stop-cock for the water supply of the whole block.

Mary wasn't at all keen on the idea of pain-killers or anti-tetanus injections, but I insisted, if only to restore my own faith in my practical abilities. I trundled her off to hospital, pale and protesting, and arranged for a speedy injection of 'Fortral'. Her pain visibly subsided, and I was just beginning to feel pleased with myself when she became incoherent and began to stagger about. I recognise certain days when things can go wrong, and it seemed best to take her straight home. Her husband turned out to be a very large bearded sailor, and his expression as I supported his 'drunken' bandaged wife over the threshold of their bedroom was memorable. I could see that neither of them was feeling well so I didn't stay long.

